# Crystal structure of dilithium biphenyl-4,4′-di­sulfonate dihydrate

**DOI:** 10.1107/S2056989023010411

**Published:** 2024-01-01

**Authors:** Hitoshi Kumagai, Satoshi Kawata, Nobuhiro Ogihara

**Affiliations:** a Nagakute, Aichi 480-1192, Japan; b Jonan-ku, Fukuoka 814-0180, Japan; Tokyo University of Science, Japan

**Keywords:** crystal structure, hydrogen bonding, Li ion

## Abstract

The asymmetric unit of the title compound, μ-biphenyl-4,4′-di­sulfonato-bis­(aqua­lithium), [Li_2_(C_12_H_8_O_6_S_2_)(H_2_O)_2_] or Li_2_[Bph(SO_3_)_2_](H_2_O)_2_, consists of an Li ion, half of the diphenyl-4,4′-di­sulfonate [Bph(SO_3_^−^)_2_] ligand, and a water mol­ecule. The Li ion exhibits a four-coordinate tetra­hedral geometry with three oxygen atoms of the Bph(SO_3_^−^)_2_ ligands and a water mol­ecule. The tetra­hedral LiO_4_ units, which are inter­connected by biphenyl moieties, form a layer structure parallel to (100). These layers are further connected by hydrogen-bonding inter­actions to yield a three-dimensional network.

## Abstract 2

The asymmetric unit of the title compound consists of an Li ion, half of the diphenyl-4,4-di­sulfonate ligand, and a water mol­ecule. The Li ion exhibits a four-coordinate tetra­hedral geometry with three oxygen atoms of the Bph(SO_3_^−^)_2_ ligands and a water mol­ecule. The tetra­hedral LiO_4_ units, which are inter­connected by biphenyl moieties, form a layer structure parallel to (100). These layers are further connected by hydrogen-bonding inter­actions to yield a three-dimensional network.

## 1. Chemical context

Coordination networks (CNs) are crystalline materials composed of infinite arrays of *s*-block metal ions, connected by organic linkers, forming chain, layer or 3-D networks. These materials offer several advantages such as being non-toxic, abundant on the planet, and cheap and provide good results when gravimetric methods are used (Banerjee & Parise 2011 ▸). Li–di­carboxyl­ates may be good candidates as electrode materials for eco-friendly alternatives to other inorganic materials, and have been reported for use in battery applications (Armand *et al.*, 2009 ▸; Ogihara *et al.*, 2014 ▸, 2023 ▸; Yasuda & Ogihara, 2014 ▸; Mikita *et al.*, 2020 ▸). To improve our chemistry and electrode applications, we investigated CNs using di­sulfonate ligands. While the structures of di­carboxyl­ate salts of alkali metals have been reported (Banerjee & Parise, 2011 ▸), the CNs of the di­sulfonates of alkali metals are still scarcely reported. Our present investigation focuses on the use of diphenyl-4,4′-di­sulfonic acid [Bph(SO_3_H)_2_] as a structural building block in the synthesis of CNs. Here, we report a rare example of a crystal structure of a Li–di­sulfonate CN material. 
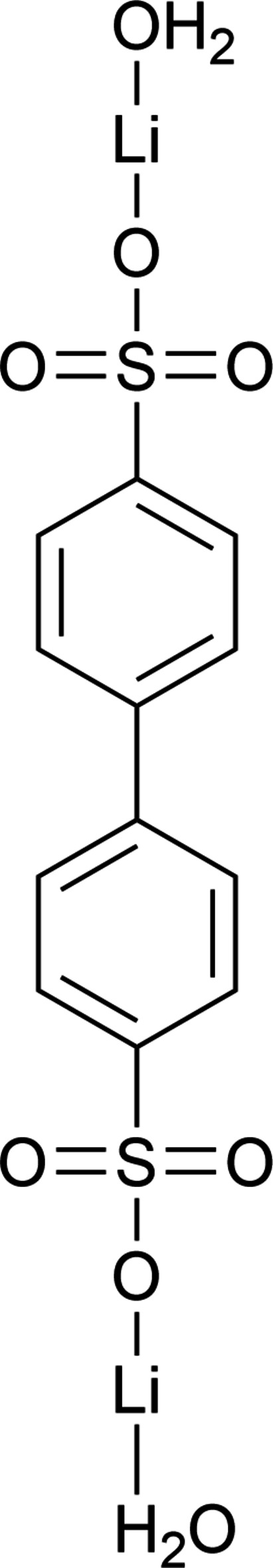


## 2. Structural commentary

**Figure 1:**
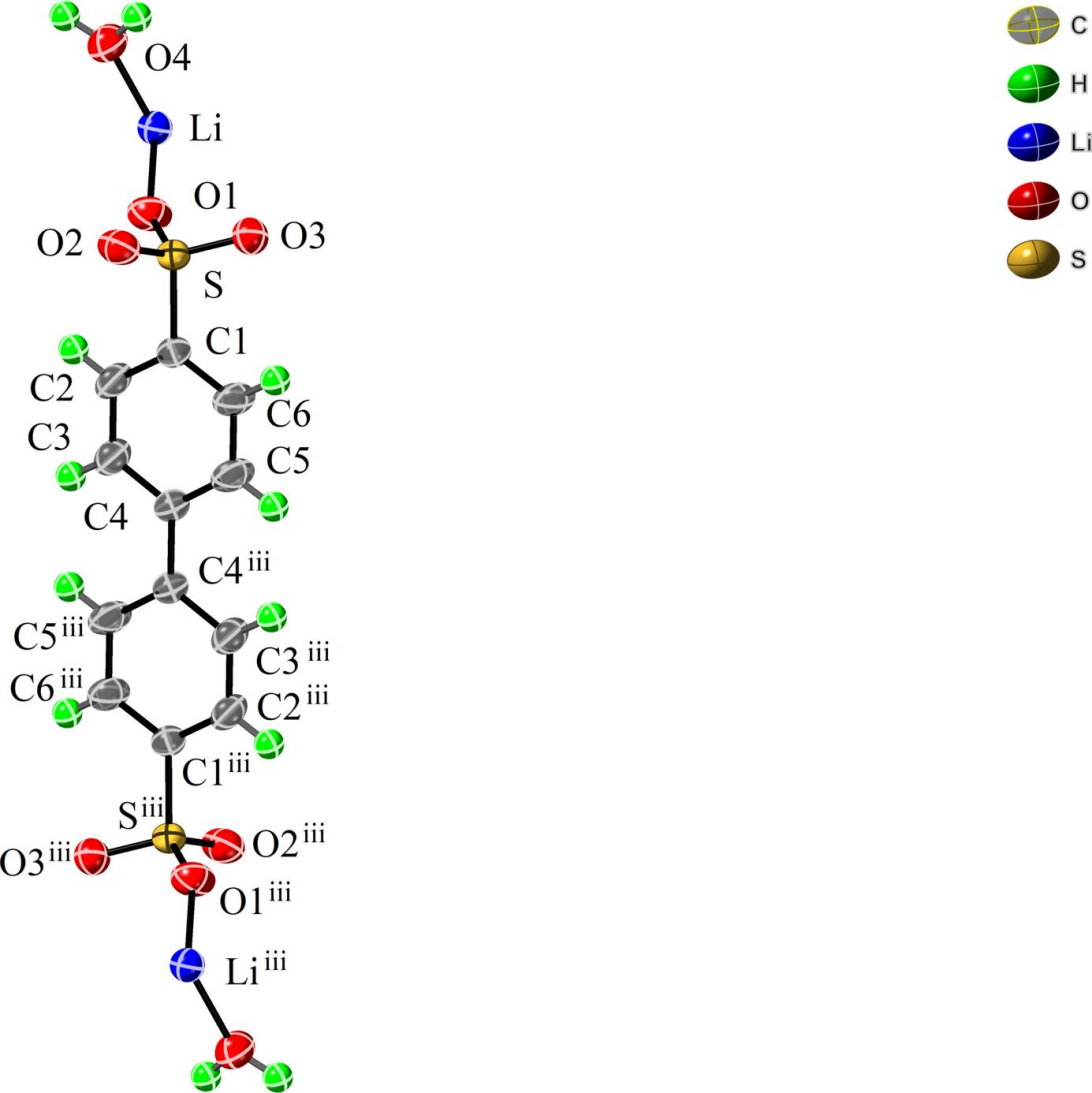
Part of the crystal structure of the title compound with labeling scheme and 50% probability displacement ellipsoids. [Symmetry code: (iii) −*x* + 2, −*y* + 1, −*z* + 1.]

**Figure 2:**
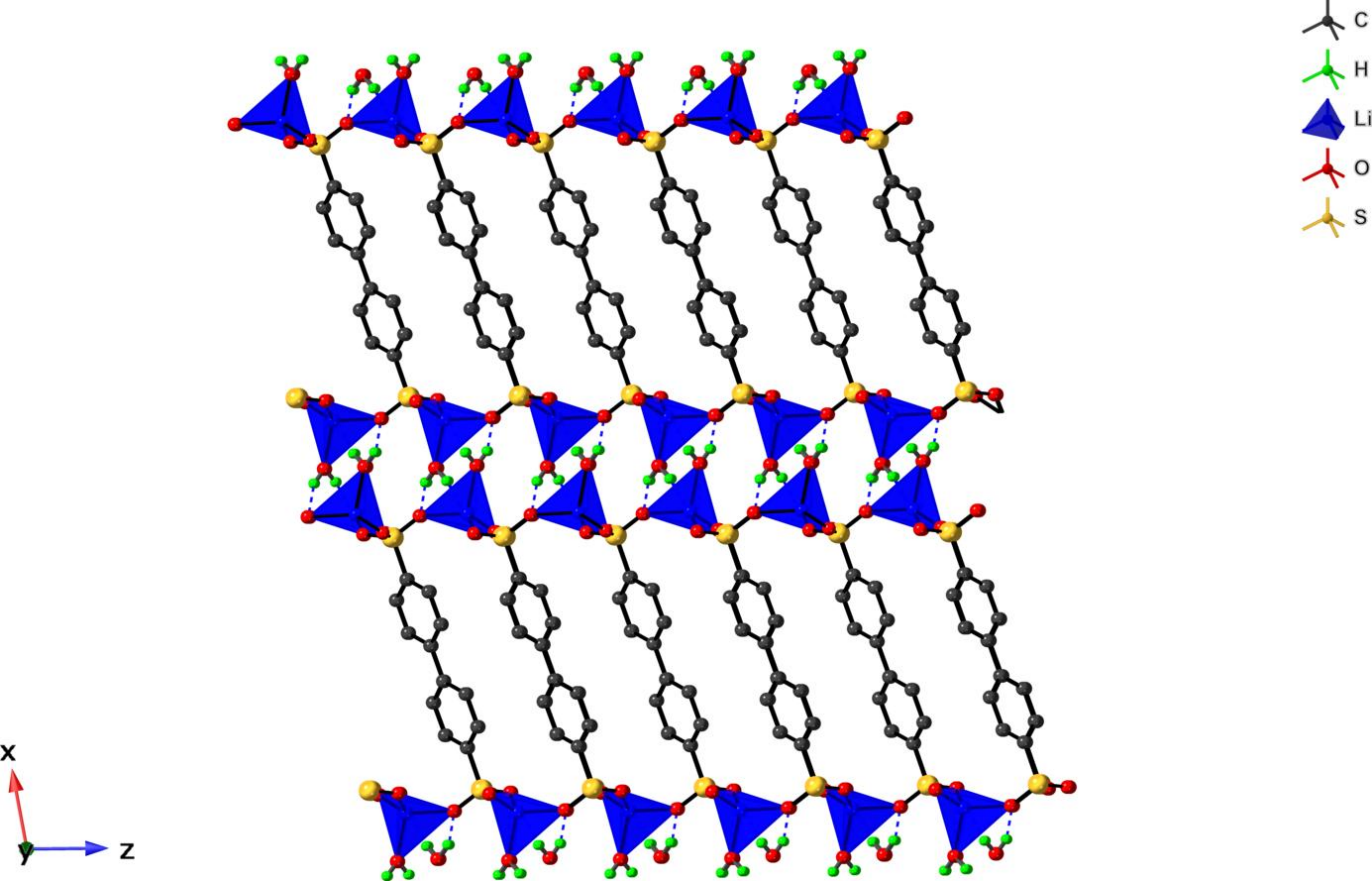
View of the layer structure of the title compound along the crystallographic *b*-axis. The layer is built up by LiO_4_ tetra­hedra connected by the organic ligands. The dashed lines represent hydrogen bonds between the oxygen atoms of the Bph(SO_3_^−^)_2_ ligands and the coordinated water mol­ecules.

**Figure 3:**
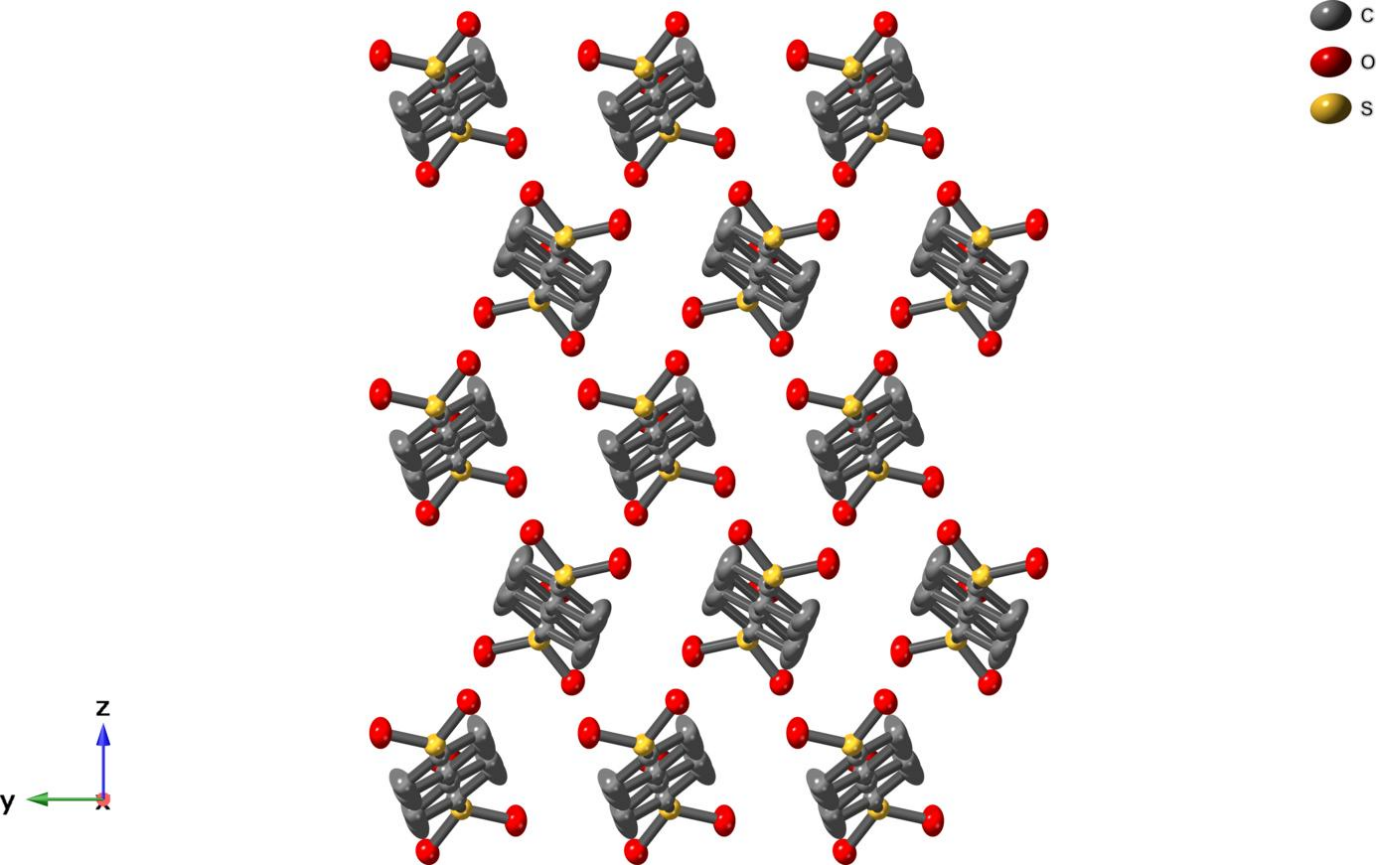
View of the herringbone-type stacking structure in the layer along the crystallographic *a*-axis.

The title compound [Li_2_(Bph(SO_3_)_2_)(H_2_O)_2_] (Fig. 1 ▸) consists of two Li cations, two water mol­ecules, and a diphenyl-4,4′-di­sulfonate [Bph(SO_3_^−^)_2_] ligand. Its asymmetric unit consists of an Li ion, half of the Bph(SO_3_^−^)_2_ ligand, and a water mol­ecule. The key feature of the structure is a di-periodic layer structure in which the layers are built up by LiO_4_ units bridged by Bph(SO_3_^−^)_2_ ligands (Fig. 2 ▸). The biphenyl groups of the ligands exhibit a planar and herringbone-type arrangement in the layer (Fig. 3 ▸). Two parallel biphenyl groups are stacked not in a face-to-face but rather in a parallel-displaced fashion. The slippage of the layers is 4.43 Å and the nearest inter­molecular centroid-to-centroid distance between adjacent parallel phenyl groups is 5.47 Å. The angle formed by the two centroids of the phenyl rings and the ring plane is 34.5°. Inter­molecular distances between the carbon atoms of the planar biphenyl moieties of 3.66 Å are indicative of some degree of π–π stacking inter­action along the crystallographic *b*-axis direction. Similar herringbone-type stacking of aromatic organic moieties are found in Li–di­carboxyl­ate CN materials in which herringbone-type stacking structures play an important role in electron mobilities and electrode performance (Ogihara *et al.*, 2017 ▸; Ozawa *et al.*, 2018 ▸). The Li cation exhibits a four-coordinate tetra­hedral geometry formed by an oxygen atom of a coordinated water mol­ecule and three oxygen atoms coming from three different Bph(SO_3_^−^)_2_ ligands. The tetra­hedrons are connected to one another by O–S–O bridges of the di­sulfonate group, and the shortest Li⋯Li distance is 4.80 Å. All the oxygen atoms of a sulfonate group coordinate to different Li cations. Thus, each sulfonate group coordinates to three Li cations to obtain a di-periodic layer. The bond distances between the Li cation and the oxygen atoms lie in the range 1.901 (5)–1.944 (5) Å at angles of 103.7 (2)–114.8 (2) °, which are shorter than those of reported Na_2_-di­sulfonate [2.313 (3)–2.560 (3) Å] and K_2_-di­sulfonate [2.657 (3)–3.079 (4) Å] complexes (Albat & Stock 2016 ▸; Smith *et al.*, 2007 ▸). Similar trends of bond distances are observed in alkali metal–carboxyl­ate network materials (Banerjee & Parise, 2011 ▸).

## 3. Supra­molecular features

The hydrogen atoms of the coordinated water mol­ecules are oriented in such a direction exiting the di-periodic layers to form hydrogen-bonding inter­actions (Table 1 ▸). A hydrogen atom of the water mol­ecule (H4) and an oxygen atom of the Bph(SO_3_^−^)_2_ ligand acts as a hydrogen-bond donor and a hydrogen-bond acceptor, respectively, resulting in a three-dimensional hydrogen-bonding network (Fig. 2 ▸). Because of the hydrogen-bonding inter­action, another hydrogen atom of the coordinated water mol­ecule (H1) is directed towards the oxygen atom of the Bph(SO_3_^−^)_2_ ligand, where the distance between the oxygen atoms of 3.204 (3) Å is indicative of some degree of inter­action. Li_2_–di­carboxyl­ates where the di­carboxyl­ate is terephthalate, biphenyl di­carboxyl­ate or naphthalene di­carboxyl­ate, also consist of LiO_4_ layers (Banerjee & Parise 2011 ▸; Kaduk *et al.*, 2000 ▸; Armand *et al.*, 2009 ▸; Banerjee *et al.*, 2009*a* ▸,*b* ▸; Ogihara *et al.*, 2014 ▸). In contrast to the sulfonate compound, four oxygen atoms come from the carboxyl­ate group and LiO_4_ units share the edges and corners of the tetra­hedrons, forming a coordination-bonded three-dimensional structure in these Li_2_–di­carboxyl­ates.

## 4. Database survey

A survey of the Cambridge Structural Database (CSD, v5.44, April 2023; Groom *et al.*, 2016 ▸) for structures with biphenyl and sulfonate and alkali metals resulted in seven hits. Of these, the alkali metal-coordinated compounds are a potassium complex (HIQKEY; Smith *et al.*, 2007 ▸), which is related to this work, and a sodium complex (SIWVUP; Anderson *et al.*, 1998 ▸). No coordination bonds are found in other alkali-metal salts. Our structure is a rare example of the crystal structure of an Li–di­sulfonate CN material.

## 5. Synthesis and crystallization

An aqueous solution (5 mL) of LiOH (0.28 g, 1 mmol L^−1^) was added to an aqueous solution of Bph(SO_3_H)_2_ (1.8 g, 2 mmol L^−1^). Colorless crystals began to form at ambient temperature in one month. One of these crystals was used for X-ray crystallography.

## 6. Refinement

Crystal data, data collection and structure refinement details are summarized in Table 2 ▸. Hydrogen-atom parameters were fully refined. The final cycle of the full-matrix least-squares refinement on *F* ^2^ was based on 1666 observed reflections and 133 variable parameters.

## Supplementary Material

Crystal structure: contains datablock(s) global, I. DOI: 10.1107/S2056989023010411/jp2001sup1.cif

Structure factors: contains datablock(s) I. DOI: 10.1107/S2056989023010411/jp2001Isup2.hkl

Supporting information file. DOI: 10.1107/S2056989023010411/jp2001Isup3.cdx

CCDC reference: 2295223

Additional supporting information:  crystallographic information; 3D view; checkCIF report

## Figures and Tables

**Table 1:** Hydrogen-bond geometry (Å, °)

*D*—H⋯*A*	*D*—H	H⋯*A*	*D*⋯*A*	*D*—H⋯*A*
O4—H4⋯O2^i^	0.89 (5)	2.17 (5)	3.016 (3)	157 (5)
O4—H1⋯O3^ii^	2.41 (5)	3.21 (1)	0.89 (5)	149 (4)
O4—H1⋯O4^iii^	2.50 (5)	3.14 (1)	0.89 (5)	129 (4)

Symmetry codes: (i) 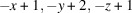 ; (ii) 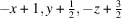 ; (iii) 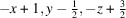 .

**Table 2:** Experimental details

Crystal data	
Chemical formula	[Li_2_(C_12_H_8_O_6_S_2_)(H_2_O)_2_]
*M*_r_	362.22
Crystal system, space group	Monoclinic, *P*2_1_/*c*
Temperature (K)	286
*a*, *b*, *c* (Å)	15.8584 (11), 5.3693 (4), 8.8636 (6)
β (°)	99.994 (7)
*V* (Å^3^)	743.27 (9)
*Z*	2
Radiation type	Mo *K*α
μ (mm^−1^)	0.40
Crystal size (mm)	0.50 × 0.40 × 0.20

Data collection	
Diffractometer	Rigaku R-AXIS RAPID
Absorption correction	Multi-scan (*ABSCOR*; Rigaku, 1995 ▸)
*T*_min_, *T*_max_	0.213, 0.924
No. of measured, independent and observed [*F* ^2^ > 2.0σ(*F* ^2^)] reflections	9858, 1666, 1490
*R*_int_	0.067
(sin θ/λ)_max_ (Å^−1^)	0.649

Refinement	
*R*[*F* ^2^ > 2σ(*F* ^2^)], *wR*(*F* ^2^), *S*	0.061, 0.159, 1.12
No. of reflections	1666
No. of parameters	133
No. of restraints	3
H-atom treatment	All H-atom parameters refined
Δρ_max_, Δρ_min_ (e Å^−3^)	0.74, −0.28

Computer programs: *RAPID-AUTO* (Rigaku, 1995 ▸), *SHELXT2014/5* (Sheldrick, 2015*a* ▸), *SHELXL2018/3* (Sheldrick, 2015*b* ▸) and *CrystalStructure* (Rigaku, 2019 ▸).

## supplementary crystallographic information

### Crystal data

**Table:**

[Li_2_(C_12_H_8_O_6_S_2_)(H_2_O)_2_]	*F*(000) = 372.00
*M**_r_* = 362.22	*D*_x_ = 1.618 Mg m^−^^3^
Monoclinic, *P*2_1_/*c*	Mo *K*α radiation, λ = 0.71075 Å
*a* = 15.8584 (11) Å	Cell parameters from 17604 reflections
*b* = 5.3693 (4) Å	θ = 3.2–27.6°
*c* = 8.8636 (6) Å	µ = 0.40 mm^−^^1^
β = 99.994 (7)°	*T* = 286 K
*V* = 743.27 (9) Å^3^	Block, colorless
*Z* = 2	0.50 × 0.40 × 0.20 mm

### Data collection

**Table:**

Rigaku R-AXIS RAPID diffractometer	1490 reflections with *F*^2^ > 2.0σ(*F*^2^)
Detector resolution: 10.000 pixels mm^-1^	*R*_int_ = 0.067
ω scans	θ_max_ = 27.5°, θ_min_ = 3.9°
Absorption correction: multi-scan (ABSCOR; Rigaku, 1995)	*h* = −20→20
*T*_min_ = 0.213, *T*_max_ = 0.924	*k* = −6→6
9858 measured reflections	*l* = −11→11
1666 independent reflections	

### Refinement

**Table:**

Refinement on *F*^2^	Secondary atom site location: difference Fourier map
*R*[*F*^2^ > 2σ(*F*^2^)] = 0.061	Hydrogen site location: inferred from neighbouring sites
*wR*(*F*^2^) = 0.159	All H-atom parameters refined
*S* = 1.12	*w* = 1/[σ^2^(*F*_o_^2^) + (0.0948*P*)^2^ + 0.3642*P*] where *P* = (*F*_o_^2^ + 2*F*_c_^2^)/3
1666 reflections	(Δ/σ)_max_ < 0.001
133 parameters	Δρ_max_ = 0.74 e Å^−^^3^
3 restraints	Δρ_min_ = −0.28 e Å^−^^3^
Primary atom site location: structure-invariant direct methods	

### Special details

**Table:**

Geometry. All esds (except the esd in the dihedral angle between two l.s. planes) are estimated using the full covariance matrix. The cell esds are taken into account individually in the estimation of esds in distances, angles and torsion angles; correlations between esds in cell parameters are only used when they are defined by crystal symmetry. An approximate (isotropic) treatment of cell esds is used for estimating esds involving l.s. planes.
Refinement. Refinement was performed using all reflections. The weighted R-factor (wR) and goodness of fit (S) are based on F^2^. R-factor (gt) are based on F. The threshold expression of F^2^ > 2.0 sigma(F^2^) is used only for calculating R-factor (gt).

### Fractional atomic coordinates and isotropic or equivalent isotropic displacement parameters (Å^2^)

**Table:**

	*x*	*y*	*z*	*U*_iso_*/*U*_eq_	
S1	0.67846 (4)	0.56370 (10)	0.59448 (6)	0.0341 (3)	
O1	0.66681 (15)	0.8238 (3)	0.6295 (3)	0.0550 (6)	
O2	0.62416 (15)	0.4887 (5)	0.4532 (2)	0.0566 (6)	
O3	0.67041 (13)	0.4012 (4)	0.7215 (2)	0.0451 (5)	
O4	0.49748 (14)	1.0921 (5)	0.6579 (3)	0.0587 (6)	
C1	0.78551 (17)	0.5384 (4)	0.5648 (3)	0.0346 (5)	
C2	0.81715 (19)	0.7143 (6)	0.4756 (4)	0.0566 (8)	
C3	0.90075 (19)	0.6964 (6)	0.4499 (4)	0.0588 (9)	
C4	0.95484 (15)	0.5075 (4)	0.5137 (3)	0.0354 (5)	
C5	0.9221 (2)	0.3349 (6)	0.6046 (5)	0.0628 (9)	
C6	0.8381 (2)	0.3489 (6)	0.6297 (5)	0.0608 (9)	
Li1	0.6195 (3)	1.0777 (8)	0.7388 (5)	0.0413 (9)	
H1	0.464 (3)	0.986 (9)	0.696 (7)	0.13 (2)*	
H2	0.778 (3)	0.874 (10)	0.441 (5)	0.083 (13)*	
H3	0.916 (3)	0.789 (10)	0.375 (6)	0.099 (16)*	
H4	0.462 (3)	1.197 (9)	0.600 (6)	0.12 (2)*	
H5	0.954 (3)	0.196 (9)	0.644 (5)	0.078 (13)*	
H6	0.823 (3)	0.262 (9)	0.695 (6)	0.096 (16)*	

### Atomic displacement parameters (Å^2^)

**Table:**

	*U*^11^	*U*^22^	*U*^33^	*U*^12^	*U*^13^	*U*^23^
S1	0.0333 (4)	0.0345 (4)	0.0359 (4)	0.0043 (2)	0.0093 (2)	−0.0016 (2)
O1	0.0641 (13)	0.0359 (11)	0.0724 (13)	0.0116 (9)	0.0324 (11)	0.0000 (9)
O2	0.0377 (11)	0.0905 (17)	0.0413 (10)	0.0008 (10)	0.0057 (9)	−0.0115 (10)
O3	0.0481 (11)	0.0404 (9)	0.0485 (10)	−0.0001 (8)	0.0131 (8)	0.0050 (8)
O4	0.0396 (12)	0.0691 (14)	0.0666 (14)	0.0048 (10)	0.0073 (10)	0.0160 (11)
C1	0.0334 (12)	0.0316 (11)	0.0386 (11)	0.0022 (8)	0.0060 (9)	−0.0036 (8)
C2	0.0352 (13)	0.0581 (17)	0.077 (2)	0.0112 (12)	0.0120 (13)	0.0322 (15)
C3	0.0356 (14)	0.0630 (19)	0.079 (2)	0.0077 (12)	0.0137 (14)	0.0355 (16)
C4	0.0302 (13)	0.0337 (11)	0.0414 (12)	0.0007 (8)	0.0042 (10)	−0.0033 (9)
C5	0.0479 (17)	0.0465 (15)	0.100 (3)	0.0186 (13)	0.0298 (17)	0.0301 (17)
C6	0.0489 (16)	0.0460 (15)	0.095 (2)	0.0149 (13)	0.0333 (17)	0.0300 (17)
Li1	0.041 (2)	0.041 (2)	0.043 (2)	0.0029 (17)	0.0106 (18)	0.0036 (16)

### Geometric parameters (Å, º)

**Table:**

S1—O3	1.4472 (19)	C1—C2	1.381 (4)
S1—O2	1.448 (2)	C2—C3	1.387 (4)
S1—O1	1.4492 (19)	C2—H2	1.07 (5)
S1—C1	1.768 (3)	C3—C4	1.384 (4)
O1—Li1	1.901 (5)	C3—H3	0.90 (5)
O2—Li1^i^	1.922 (5)	C4—C5	1.387 (4)
O3—Li1^ii^	1.933 (5)	C4—C4^iii^	1.496 (5)
O4—Li1	1.944 (5)	C5—C6	1.390 (4)
O4—H1	0.88 (2)	C5—H5	0.94 (5)
O4—H4	0.89 (2)	C6—H6	0.81 (5)
C1—C6	1.377 (4)		
			
O3—S1—O2	112.66 (14)	C4—C3—C2	121.8 (3)
O3—S1—O1	112.48 (12)	C4—C3—H3	119 (3)
O2—S1—O1	112.00 (15)	C2—C3—H3	118 (3)
O3—S1—C1	106.61 (11)	C3—C4—C5	117.3 (2)
O2—S1—C1	107.01 (12)	C3—C4—C4^iii^	121.1 (3)
O1—S1—C1	105.51 (12)	C5—C4—C4^iii^	121.6 (3)
S1—O1—Li1	151.24 (19)	C4—C5—C6	121.5 (3)
S1—O2—Li1^i^	145.2 (2)	C4—C5—H5	121 (3)
S1—O3—Li1^ii^	134.28 (19)	C6—C5—H5	118 (3)
Li1—O4—H1	117 (4)	C1—C6—C5	120.1 (3)
Li1—O4—H4	136 (4)	C1—C6—H6	119 (4)
H1—O4—H4	106 (4)	C5—C6—H6	120 (4)
C6—C1—C2	119.4 (3)	O1—Li1—O2^iv^	114.8 (2)
C6—C1—S1	121.5 (2)	O1—Li1—O3^v^	113.3 (2)
C2—C1—S1	119.09 (19)	O2^iv^—Li1—O3^v^	107.5 (2)
C1—C2—C3	119.9 (3)	O1—Li1—O4	107.2 (3)
C1—C2—H2	117 (2)	O2^iv^—Li1—O4	103.7 (2)
C3—C2—H2	122 (2)	O3^v^—Li1—O4	109.8 (2)
			
O3—S1—O1—Li1	−27.3 (5)	O2—S1—C1—C2	74.3 (3)
O2—S1—O1—Li1	100.8 (5)	O1—S1—C1—C2	−45.2 (3)
C1—S1—O1—Li1	−143.1 (4)	C6—C1—C2—C3	1.1 (5)
O3—S1—O2—Li1^i^	−131.3 (4)	S1—C1—C2—C3	−179.5 (3)
O1—S1—O2—Li1^i^	100.7 (4)	C1—C2—C3—C4	−1.1 (6)
C1—S1—O2—Li1^i^	−14.4 (4)	C2—C3—C4—C5	0.2 (5)
O2—S1—O3—Li1^ii^	12.8 (3)	C2—C3—C4—C4^iii^	−179.3 (3)
O1—S1—O3—Li1^ii^	140.6 (3)	C3—C4—C5—C6	0.6 (6)
C1—S1—O3—Li1^ii^	−104.2 (3)	C4^iii^—C4—C5—C6	−179.8 (4)
O3—S1—C1—C6	14.5 (3)	C2—C1—C6—C5	−0.3 (6)
O2—S1—C1—C6	−106.3 (3)	S1—C1—C6—C5	−179.7 (3)
O1—S1—C1—C6	134.3 (3)	C4—C5—C6—C1	−0.6 (6)
O3—S1—C1—C2	−165.0 (2)		

Symmetry codes: (i) *x*, −*y*+3/2, *z*−1/2; (ii) *x*, *y*−1, *z*; (iii) −*x*+2, −*y*+1, −*z*+1; (iv) *x*, −*y*+3/2, *z*+1/2; (v) *x*, *y*+1, *z*.

### Hydrogen-bond geometry (Å, º)

**Table:**

*D*—H···*A*	*D*—H	H···*A*	*D*···*A*	*D*—H···*A*
O4—H4···O2^vi^	0.89 (5)	2.17 (5)	3.016 (3)	157 (5)
O4—H1···O3^vii^	2.41 (5)	3.21 (1)	0.89 (5)	149 (4)
O4—H1···O4^viii^	2.50 (5)	3.14 (1)	0.89 (5)	129 (4)

Symmetry codes: (vi) −*x*+1, −*y*+2, −*z*+1; (vii) −*x*+1, *y*+1/2, −*z*+3/2; (viii) −*x*+1, *y*−1/2, −*z*+3/2.
